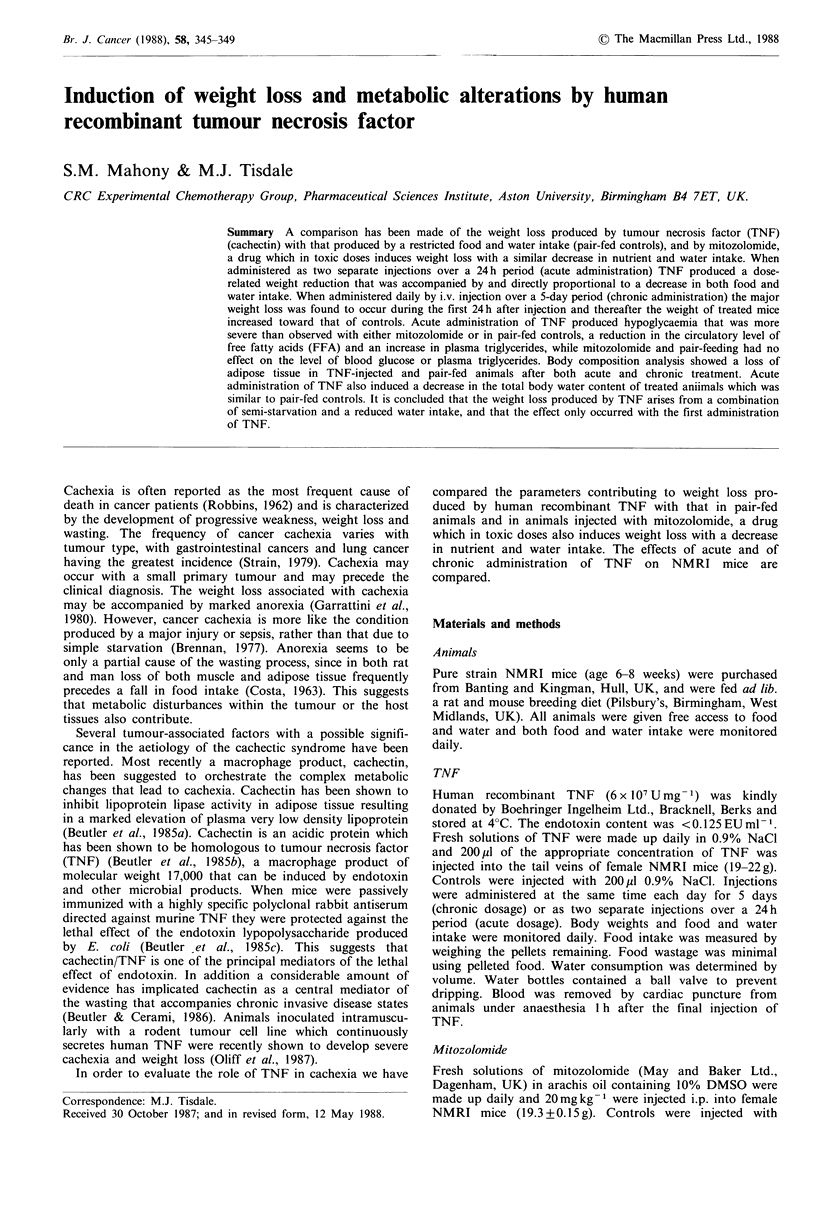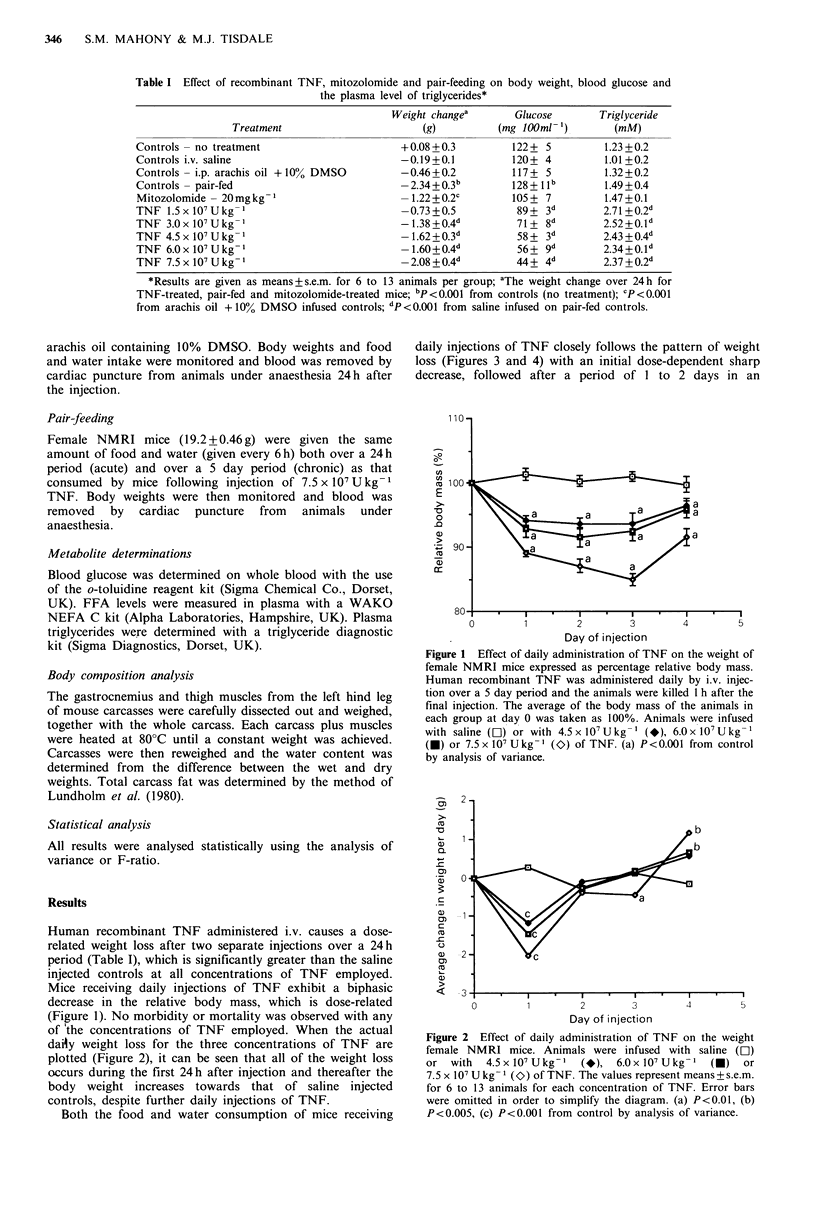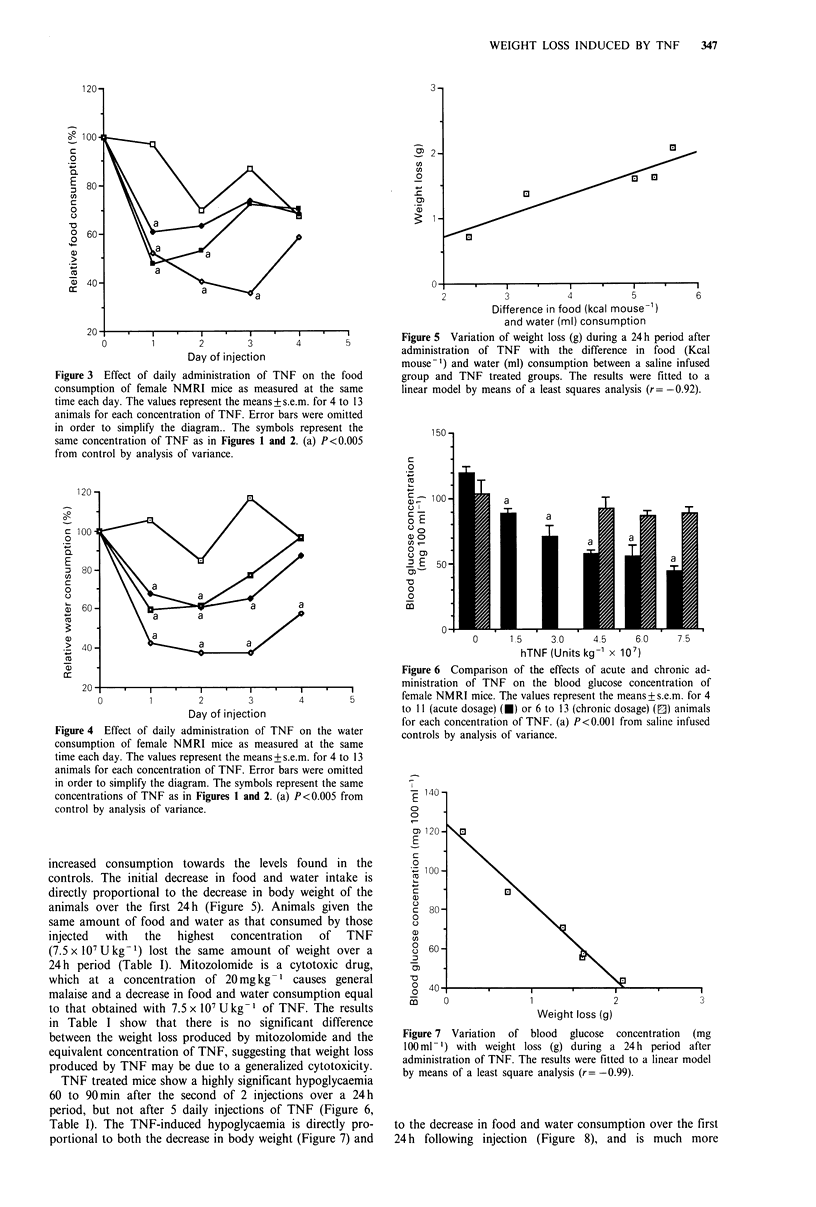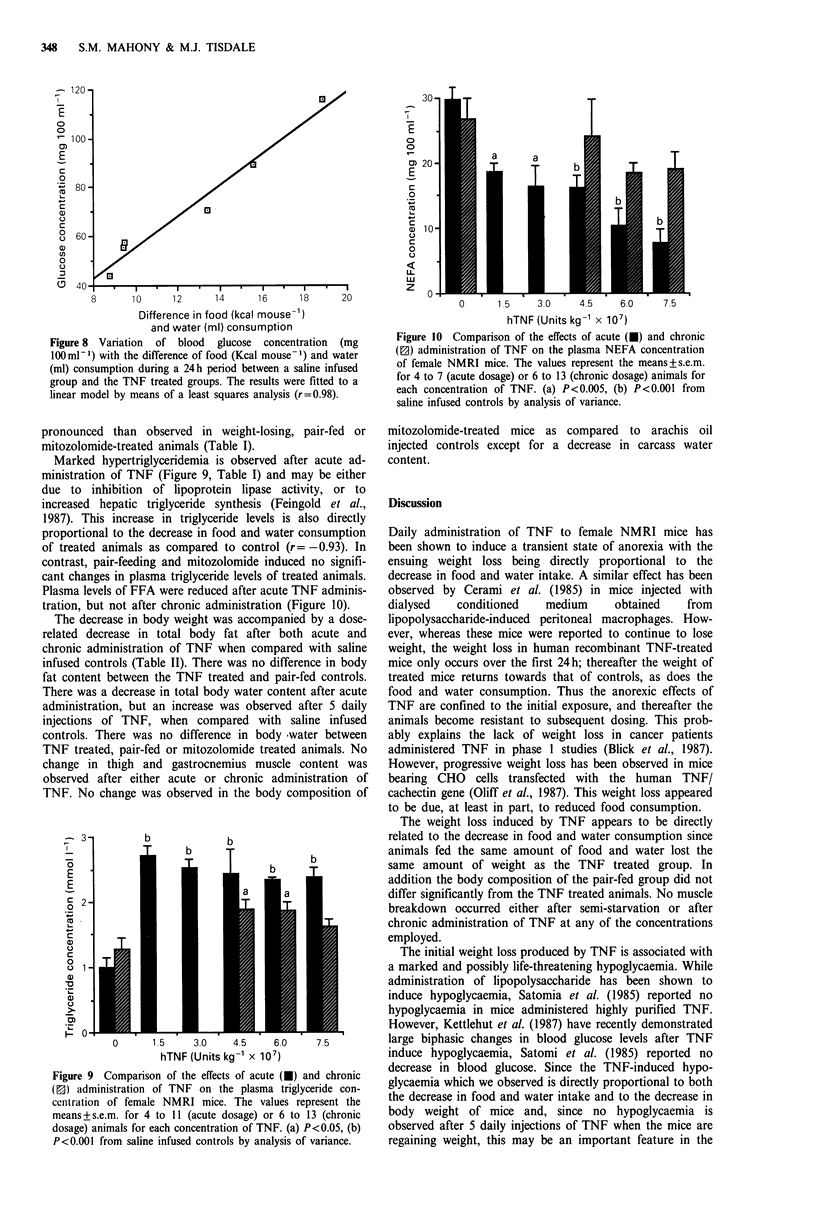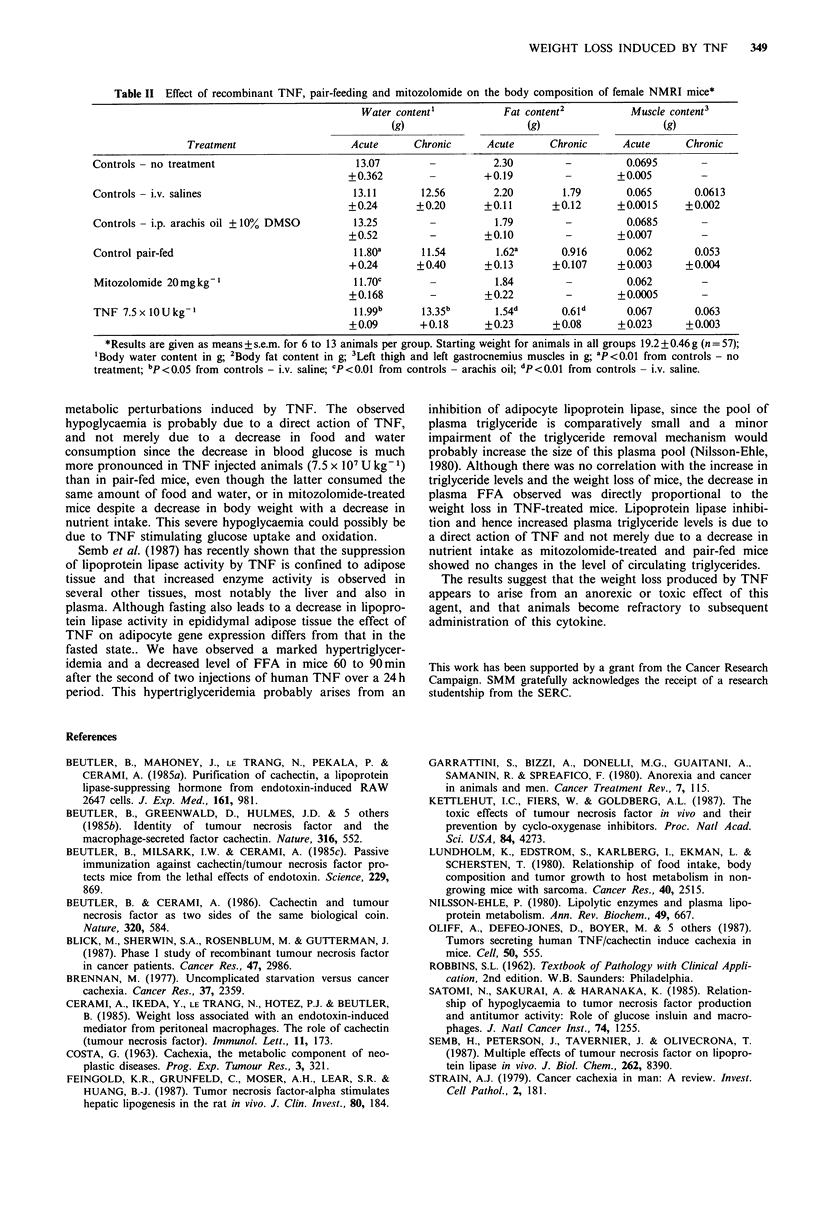# Induction of weight loss and metabolic alterations by human recombinant tumour necrosis factor.

**DOI:** 10.1038/bjc.1988.216

**Published:** 1988-09

**Authors:** S. M. Mahony, M. J. Tisdale

**Affiliations:** Pharmaceutical Sciences Institute, Aston University, Birmingham, UK.

## Abstract

A comparison has been made of the weight loss produced by tumour necrosis factor (TNF) (cachectin) with that produced by a restricted food and water intake (pair-fed controls), and by mitozolomide, a drug which in toxic doses induces weight loss with a similar decrease in nutrient and water intake. When administered as two separate injections over a 24 h period (acute administration) TNF produced a dose-related weight reduction that was accompanied by and directly proportional to a decrease in both food and water intake. When administered daily by i.v. injection over a 5-day period (chronic administration) the major weight loss was found to occur during the first 24 h after injection and thereafter the weight of treated mice increased toward that of controls. Acute administration of TNF produced hypoglycaemia that was more severe than observed with either mitozolomide or in pair-fed controls, a reduction in the circulatory level of free fatty acids (FFA) and an increase in plasma triglycerides, while mitozolomide and pair-feeding had no effect on the level of blood glucose or plasma triglycerides. Body composition analysis showed a loss of adipose tissue in TNF-injected and pair-fed animals after both acute and chronic treatment. Acute administration of TNF also induced a decrease in the total body water content of treated animals which was similar to pair-fed controls. It is concluded that the weight loss produced by TNF arises from a combination of semi-starvation and a reduced water intake, and that the effect only occurred with the first administration of TNF.


*Br. J. Cancer* (1988), **58**, 345–349 © The Macmillan Press Ltd., 1988

### Induction of weight loss and metabolic alterations by human recombinant tumour necrosis factor

S.M. Mahony & M.J. Tisdale

*CRC Experimental Chemotherapy Group, Pharmaceutical Sciences Institute, Aston University, Birmingham B4 7ET, UK.*

**Summary** A comparison has been made of the weight loss produced by tumour necrosis factor (TNF) (cachectin) with that produced by a restricted food and water intake (pair-fed controls), and by mitozolomide, a drug which in toxic doses induces weight loss with a similar decrease in nutrient and water intake. When administered as two separate injections over a 24 h period (acute administration) TNF produced a dose-related weight reduction that was accompanied by and directly proportional to a decrease in both food and water intake. When administered daily by i.v. injection over a 5-day period (chronic administration) the major weight loss was found to occur during the first 24 h after injection and thereafter the weight of treated mice increased toward that of controls. Acute administration of TNF produced hypoglycaemia that was more severe than observed with either mitozolomide or in pair-fed controls, a reduction in the circulatory level of free fatty acids (FFA) and an increase in plasma triglycerides, while mitozolomide and pair-feeding had no effect on the level of blood glucose or plasma triglycerides. Body composition analysis showed a loss of adipose tissue in TNF-injected and pair-fed animals after both acute and chronic treatment. Acute administration of TNF also induced a decrease in the total body water content of treated aniimals which was similar to pair-fed controls. It is concluded that the weight loss produced by TNF arises from a combination of semi-starvation and a reduced water intake, and that the effect only occurred with the first administration of TNF.

Cachexia is often reported as the most frequent cause of death in cancer patients (Robbins, 1962) and is characterized by the development of progressive weakness, weight loss and wasting. The frequency of cancer cachexia varies with tumour type, with gastrointestinal cancers and lung cancer having the greatest incidence (Strain, 1979). Cachexia may occur with a small primary tumour and may precede the clinical diagnosis. The weight loss associated with cachexia may be accompanied by marked anorexia (Garrattini *et al.*, 1980). However, cancer cachexia is more like the condition produced by a major injury or sepsis, rather than that due to simple starvation (Brennan, 1977). Anorexia seems to be only a partial cause of the wasting process, since in both rat and man loss of both muscle and adipose tissue frequently precedes a fall in food intake (Costa, 1963). This suggests that metabolic disturbances within the tumour or the host tissues also contribute.

Several tumour-associated factors with a possible significance in the aetiology of the cachectic syndrome have been reported. Most recently a macrophage product, cachectin, has been suggested to orchestrate the complex metabolic changes that lead to cachexia. Cachectin has been shown to inhibit lipoprotein lipase activity in adipose tissue resulting in a marked elevation of plasma very low density lipoprotein (Beutler *et al.*, 1985*a*). Cachectin is an acidic protein which has been shown to be homologous to tumour necrosis factor (TNF) (Beutler *et al.*, 1985*b*), a macrophage product of molecular weight 17,000 that can be induced by endotoxin and other microbial products. When mice were passively immunized with a highly specific polyclonal rabbit antiserum directed against murine TNF they were protected against the lethal effect of the endotoxin lypopolysaccharide produced by *E. coli* (Beutler *et al.*, 1985*c*). This suggests that cachectin/TNF is one of the principal mediators of the lethal effect of endotoxin. In addition a considerable amount of evidence has implicated cachectin as a central mediator of the wasting that accompanies chronic invasive disease states (Beutler & Cerami, 1986). Animals inoculated intramuscularly with a rodent tumour cell line which continuously secretes human TNF were recently shown to develop severe cachexia and weight loss (Oliff *et al.*, 1987).

In order to evaluate the role of TNF in cachexia we have compared the parameters contributing to weight loss produced by human recombinant TNF with that in pair-fed animals and in animals injected with mitozolomide, a drug which in toxic doses also induces weight loss with a decrease in nutrient and water intake. The effects of acute and of chronic administration of TNF on NMRI mice are compared.

Correspondence: M.J. Tisdale.
Received 30 October 1987; and in revised form, 12 May 1988.

### Materials and methods

### *Animals*

Pure strain NMRI mice (age 6–8 weeks) were purchased from Banting and Kingman, Hull, UK, and were fed *ad lib.* a rat and mouse breeding diet (Pilsbury's, Birmingham, West Midlands, UK). All animals were given free access to food and water and both food and water intake were monitored daily.

### *TNF*

Human recombinant TNF ($6 \times 10^7 U mg^{-1}$) was kindly donated by Boehringer Ingelheim Ltd., Bracknell, Berks and stored at 4°C. The endotoxin content was $<0.125 EU ml^{-1}$. Fresh solutions of TNF were made up daily in 0.9% NaCl and 200 $\mu$l of the appropriate concentration of TNF was injected into the tail veins of female NMRI mice (19–22 g). Controls were injected with 200 $\mu$l 0.9% NaCl. Injections were administered at the same time each day for 5 days (chronic dosage) or as two separate injections over a 24 h period (acute dosage). Body weights and food and water intake were monitored daily. Food intake was measured by weighing the pellets remaining. Food wastage was minimal using pelleted food. Water consumption was determined by volume. Water bottles contained a ball valve to prevent dripping. Blood was removed by cardiac puncture from animals under anaesthesia 1 h after the final injection of TNF.

### *Mitozolomide*

Fresh solutions of mitozolomide (May and Baker Ltd., Dagenham, UK) in arachis oil containing 10% DMSO were made up daily and 20 mg kg$^{-1}$ were injected i.p. into female NMRI mice ($19.3 \pm 0.15$ g). Controls were injected with

**Table I** Effect of recombinant TNF, mitozolomide and pair-feeding on body weight, blood glucose and the plasma level of triglycerides*

| Treatment | *Weight change*[a] *(g)* | *Glucose* *(mg 100ml*$^{-1}$*)* | *Triglyceride* *(mM)* |
|---|---|---|---|
| Controls – no treatment | $+0.08\pm0.3$ | $122\pm5$ | $1.23\pm0.2$ |
| Controls i.v. saline | $-0.19\pm0.1$ | $120\pm4$ | $1.01\pm0.2$ |
| Controls – i.p. arachis oil +10% DMSO | $-0.46\pm0.2$ | $117\pm5$ | $1.32\pm0.2$ |
| Controls – pair-fed | $-2.34\pm0.3$[b] | $128\pm11$[b] | $1.49\pm0.4$ |
| Mitozolomide – 20 mg kg$^{-1}$ | $-1.22\pm0.2$[c] | $105\pm7$ | $1.47\pm0.1$ |
| TNF $1.5\times10^7$ U kg$^{-1}$ | $-0.73\pm0.5$ | $89\pm3$[d] | $2.71\pm0.2$[d] |
| TNF $3.0\times10^7$ U kg$^{-1}$ | $-1.38\pm0.4$[d] | $71\pm8$[d] | $2.52\pm0.1$[d] |
| TNF $4.5\times10^7$ U kg$^{-1}$ | $-1.62\pm0.3$[d] | $58\pm3$[d] | $2.43\pm0.4$[d] |
| TNF $6.0\times10^7$ U kg$^{-1}$ | $-1.60\pm0.4$[d] | $56\pm9$[d] | $2.34\pm0.1$[d] |
| TNF $7.5\times10^7$ U kg$^{-1}$ | $-2.08\pm0.4$[d] | $44\pm4$[d] | $2.37\pm0.2$[d] |

*Results are given as means ± s.e.m. for 6 to 13 animals per group; [a]The weight change over 24 h for TNF-treated, pair-fed and mitozolomide-treated mice; [b]$P<0.001$ from controls (no treatment); [c]$P<0.001$ from arachis oil +10% DMSO infused controls; [d]$P<0.001$ from saline infused on pair-fed controls.

arachis oil containing 10% DMSO. Body weights and food and water intake were monitored and blood was removed by cardiac puncture from animals under anaesthesia 24 h after the injection.

*Pair-feeding*

Female NMRI mice (19.2 ± 0.46 g) were given the same amount of food and water (given every 6 h) both over a 24 h period (acute) and over a 5 day period (chronic) as that consumed by mice following injection of $7.5\times10^7$ U kg$^{-1}$ TNF. Body weights were then monitored and blood was removed by cardiac puncture from animals under anaesthesia.

*Metabolite determinations*

Blood glucose was determined on whole blood with the use of the *o*-toluidine reagent kit (Sigma Chemical Co., Dorset, UK). FFA levels were measured in plasma with a WAKO NEFA C kit (Alpha Laboratories, Hampshire, UK). Plasma triglycerides were determined with a triglyceride diagnostic kit (Sigma Diagnostics, Dorset, UK).

*Body composition analysis*

The gastrocnemius and thigh muscles from the left hind leg of mouse carcasses were carefully dissected out and weighed, together with the whole carcass. Each carcass plus muscles were heated at 80°C until a constant weight was achieved. Carcasses were then reweighed and the water content was determined from the difference between the wet and dry weights. Total carcass fat was determined by the method of Lundholm *et al.* (1980).

*Statistical analysis*

All results were analysed statistically using the analysis of variance or F-ratio.

### Results

Human recombinant TNF administered i.v. causes a dose-related weight loss after two separate injections over a 24 h period (Table I), which is significantly greater than the saline injected controls at all concentrations of TNF employed. Mice receiving daily injections of TNF exhibit a biphasic decrease in the relative body mass, which is dose-related (Figure 1). No morbidity or mortality was observed with any of the concentrations of TNF employed. When the actual daily weight loss for the three concentrations of TNF are plotted (Figure 2), it can be seen that all of the weight loss occurs during the first 24 h after injection and thereafter the body weight increases towards that of saline injected controls, despite further daily injections of TNF.

Both the food and water consumption of mice receiving daily injections of TNF closely follows the pattern of weight loss (Figures 3 and 4) with an initial dose-dependent sharp decrease, followed after a period of 1 to 2 days in an

**Figure 1** Effect of daily administration of TNF on the weight of female NMRI mice expressed as percentage relative body mass. Human recombinant TNF was administered daily by i.v. injection over a 5 day period and the animals were killed 1 h after the final injection. The average of the body mass of the animals in each group at day 0 was taken as 100%. Animals were infused with saline (□) or with $4.5\times10^7$ U kg$^{-1}$ (◆), $6.0\times10^7$ U kg$^{-1}$ (■) or $7.5\times10^7$ U kg$^{-1}$ (◇) of TNF. (a) $P<0.001$ from control by analysis of variance.

**Figure 2** Effect of daily administration of TNF on the weight female NMRI mice. Animals were infused with saline (□) or with $4.5\times10^7$ U kg$^{-1}$ (◆), $6.0\times10^7$ U kg$^{-1}$ (■) or $7.5\times10^7$ U kg$^{-1}$ (◇) of TNF. The values represent means ± s.e.m. for 6 to 13 animals for each concentration of TNF. Error bars were omitted in order to simplify the diagram. (a) $P<0.01$, (b) $P<0.005$, (c) $P<0.001$ from control by analysis of variance.

**Figure 3** Effect of daily administration of TNF on the food consumption of female NMRI mice as measured at the same time each day. The values represent the means ± s.e.m. for 4 to 13 animals for each concentration of TNF. Error bars were omitted in order to simplify the diagram.. The symbols represent the same concentration of TNF as in **Figures 1 and 2**. (a) $P<0.005$ from control by analysis of variance.

**Figure 4** Effect of daily administration of TNF on the water consumption of female NMRI mice as measured at the same time each day. The values represent the means ± s.e.m. for 4 to 13 animals for each concentration of TNF. Error bars were omitted in order to simplify the diagram. The symbols represent the same concentrations of TNF as in **Figures 1 and 2**. (a) $P<0.005$ from control by analysis of variance.

increased consumption towards the levels found in the controls. The initial decrease in food and water intake is directly proportional to the decrease in body weight of the animals over the first 24 h (Figure 5). Animals given the same amount of food and water as that consumed by those injected with the highest concentration of TNF ($7.5 \times 10^7$ U kg$^{-1}$) lost the same amount of weight over a 24 h period (Table I). Mitozolomide is a cytotoxic drug, which at a concentration of 20 mg kg$^{-1}$ causes general malaise and a decrease in food and water consumption equal to that obtained with $7.5 \times 10^7$ U kg$^{-1}$ of TNF. The results in Table I show that there is no significant difference between the weight loss produced by mitozolomide and the equivalent concentration of TNF, suggesting that weight loss produced by TNF may be due to a generalized cytotoxicity.

TNF treated mice show a highly significant hypoglycaemia 60 to 90 min after the second of 2 injections over a 24 h period, but not after 5 daily injections of TNF (Figure 6, Table I). The TNF-induced hypoglycaemia is directly proportional to both the decrease in body weight (Figure 7) and

**Figure 5** Variation of weight loss (g) during a 24 h period after administration of TNF with the difference in food (Kcal mouse$^{-1}$) and water (ml) consumption between a saline infused group and TNF treated groups. The results were fitted to a linear model by means of a least squares analysis ($r = -0.92$).

**Figure 6** Comparison of the effects of acute and chronic administration of TNF on the blood glucose concentration of female NMRI mice. The values represent the means ± s.e.m. for 4 to 11 (acute dosage) (■) or 6 to 13 (chronic dosage) (▨) animals for each concentration of TNF. (a) $P<0.001$ from saline infused controls by analysis of variance.

**Figure 7** Variation of blood glucose concentration (mg 100 ml$^{-1}$) with weight loss (g) during a 24 h period after administration of TNF. The results were fitted to a linear model by means of a least square analysis ($r = -0.99$).

to the decrease in food and water consumption over the first 24 h following injection (Figure 8), and is much more

**Figure 8** Variation of blood glucose concentration (mg 100 ml$^{-1}$) with the difference of food (Kcal mouse$^{-1}$) and water (ml) consumption during a 24 h period between a saline infused group and the TNF treated groups. The results were fitted to a linear model by means of a least squares analysis ($r=0.98$).

**Figure 10** Comparison of the effects of acute (■) and chronic (▨) administration of TNF on the plasma NEFA concentration of female NMRI mice. The values represent the means±s.e.m. for 4 to 7 (acute dosage) or 6 to 13 (chronic dosage) animals for each concentration of TNF. (a) $P<0.005$, (b) $P<0.001$ from saline infused controls by analysis of variance.

pronounced than observed in weight-losing, pair-fed or mitozolomide-treated animals (Table I).

Marked hypertriglyceridemia is observed after acute administration of TNF (Figure 9, Table I) and may be either due to inhibition of lipoprotein lipase activity, or to increased hepatic triglyceride synthesis (Feingold *et al.*, 1987). This increase in triglyceride levels is also directly proportional to the decrease in food and water consumption of treated animals as compared to control ($r=-0.93$). In contrast, pair-feeding and mitozolomide induced no significant changes in plasma triglyceride levels of treated animals. Plasma levels of FFA were reduced after acute TNF administration, but not after chronic administration (Figure 10).

The decrease in body weight was accompanied by a dose-related decrease in total body fat after both acute and chronic administration of TNF when compared with saline infused controls (Table II). There was no difference in body fat content between the TNF treated and pair-fed controls. There was a decrease in total body water content after acute administration, but an increase was observed after 5 daily injections of TNF, when compared with saline infused controls. There was no difference in body water between TNF treated, pair-fed or mitozolomide treated animals. No change in thigh and gastrocnemius muscle content was observed after either acute or chronic administration of TNF. No change was observed in the body composition of mitozolomide-treated mice as compared to arachis oil injected controls except for a decrease in carcass water content.

**Figure 9** Comparison of the effects of acute (■) and chronic (▨) administration of TNF on the plasma triglyceride concentration of female NMRI mice. The values represent the means±s.e.m. for 4 to 11 (acute dosage) or 6 to 13 (chronic dosage) animals for each concentration of TNF. (a) $P<0.05$, (b) $P<0.001$ from saline infused controls by analysis of variance.

### Discussion

Daily administration of TNF to female NMRI mice has been shown to induce a transient state of anorexia with the ensuing weight loss being directly proportional to the decrease in food and water intake. A similar effect has been observed by Cerami *et al.* (1985) in mice injected with dialysed conditioned medium obtained from lipopolysaccharide-induced peritoneal macrophages. However, whereas these mice were reported to continue to lose weight, the weight loss in human recombinant TNF-treated mice only occurs over the first 24 h; thereafter the weight of treated mice returns towards that of controls, as does the food and water consumption. Thus the anorexic effects of TNF are confined to the initial exposure, and thereafter the animals become resistant to subsequent dosing. This probably explains the lack of weight loss in cancer patients administered TNF in phase 1 studies (Blick *et al.*, 1987). However, progressive weight loss has been observed in mice bearing CHO cells transfected with the human TNF/cachectin gene (Oliff *et al.*, 1987). This weight loss appeared to be due, at least in part, to reduced food consumption.

The weight loss induced by TNF appears to be directly related to the decrease in food and water consumption since animals fed the same amount of food and water lost the same amount of weight as the TNF treated group. In addition the body composition of the pair-fed group did not differ significantly from the TNF treated animals. No muscle breakdown occurred either after semi-starvation or after chronic administration of TNF at any of the concentrations employed.

The initial weight loss produced by TNF is associated with a marked and possibly life-threatening hypoglycaemia. While administration of lipopolysaccharide has been shown to induce hypoglycaemia, Satomia *et al.* (1985) reported no hypoglycaemia in mice administered highly purified TNF. However, Kettlehut *et al.* (1987) have recently demonstrated large biphasic changes in blood glucose levels after TNF induce hypoglycaemia, Satomi *et al.* (1985) reported no decrease in blood glucose. Since the TNF-induced hypoglycaemia which we observed is directly proportional to both the decrease in food and water intake and to the decrease in body weight of mice and, since no hypoglycaemia is observed after 5 daily injections of TNF when the mice are regaining weight, this may be an important feature in the

**Table II** Effect of recombinant TNF, pair-feeding and mitozolomide on the body composition of female NMRI mice*

| Treatment | *Water content*[1] (g) Acute | *Water content*[1] (g) Chronic | *Fat content*[2] (g) Acute | *Fat content*[2] (g) Chronic | *Muscle content*[3] (g) Acute | *Muscle content*[3] (g) Chronic |
|---|---|---|---|---|---|---|
| Controls – no treatment | 13.07 ±0.362 | – – | 2.30 +0.19 | – – | 0.0695 ±0.005 | – – |
| Controls – i.v. salines | 13.11 ±0.24 | 12.56 ±0.20 | 2.20 ±0.11 | 1.79 ±0.12 | 0.065 ±0.0015 | 0.0613 ±0.002 |
| Controls – i.p. arachis oil ±10% DMSO | 13.25 ±0.52 | – – | 1.79 ±0.10 | – – | 0.0685 ±0.007 | – – |
| Control pair-fed | 11.80[a] +0.24 | 11.54 ±0.40 | 1.62[a] ±0.13 | 0.916 ±0.107 | 0.062 ±0.003 | 0.053 ±0.004 |
| Mitozolomide 20 mg kg$^{-1}$ | 11.70[c] ±0.168 | – – | 1.84 ±0.22 | – – | 0.062 ±0.0005 | – – |
| TNF 7.5 × 10 U kg$^{-1}$ | 11.99[b] ±0.09 | 13.35[b] +0.18 | 1.54[d] ±0.23 | 0.61[d] ±0.08 | 0.067 ±0.023 | 0.063 ±0.003 |

*Results are given as means ± s.e.m. for 6 to 13 animals per group. Starting weight for animals in all groups 19.2 ± 0.46 g ($n = 57$); [1]Body water content in g; [2]Body fat content in g; [3]Left thigh and left gastrocnemius muscles in g; [a]$P < 0.01$ from controls – no treatment; [b]$P < 0.05$ from controls – i.v. saline; [c]$P < 0.01$ from controls – arachis oil; [d]$P < 0.01$ from controls – i.v. saline.

metabolic perturbations induced by TNF. The observed hypoglycaemia is probably due to a direct action of TNF, and not merely due to a decrease in food and water consumption since the decrease in blood glucose is much more pronounced in TNF injected animals (7.5 × 10$^7$ U kg$^{-1}$) than in pair-fed mice, even though the latter consumed the same amount of food and water, or in mitozolomide-treated mice despite a decrease in body weight with a decrease in nutrient intake. This severe hypoglycaemia could possibly be due to TNF stimulating glucose uptake and oxidation.

Semb *et al.* (1987) has recently shown that the suppression of lipoprotein lipase activity by TNF is confined to adipose tissue and that increased enzyme activity is observed in several other tissues, most notably the liver and also in plasma. Although fasting also leads to a decrease in lipoprotein lipase activity in epididymal adipose tissue the effect of TNF on adipocyte gene expression differs from that in the fasted state.. We have observed a marked hypertriglyceridemia and a decreased level of FFA in mice 60 to 90 min after the second of two injections of human TNF over a 24 h period. This hypertriglyceridemia probably arises from an inhibition of adipocyte lipoprotein lipase, since the pool of plasma triglyceride is comparatively small and a minor impairment of the triglyceride removal mechanism would probably increase the size of this plasma pool (Nilsson-Ehle, 1980). Although there was no correlation with the increase in triglyceride levels and the weight loss of mice, the decrease in plasma FFA observed was directly proportional to the weight loss in TNF-treated mice. Lipoprotein lipase inhibition and hence increased plasma triglyceride levels is due to a direct action of TNF and not merely due to a decrease in nutrient intake as mitozolomide-treated and pair-fed mice showed no changes in the level of circulating triglycerides.

The results suggest that the weight loss produced by TNF appears to arise from an anorexic or toxic effect of this agent, and that animals become refractory to subsequent administration of this cytokine.

This work has been supported by a grant from the Cancer Research Campaign. SMM gratefully acknowledges the receipt of a research studentship from the SERC.

### References

BEUTLER, B., MAHONEY, J., LE TRANG, N., PEKALA, P. & CERAMI, A. (1985*a*). Purification of cachectin, a lipoprotein lipase-suppressing hormone from endotoxin-induced RAW 2647 cells. *J. Exp. Med.*, **161**, 981.

BEUTLER, B., GREENWALD, D., HULMES, J.D. & 5 others (1985*b*). Identity of tumour necrosis factor and the macrophage-secreted factor cachectin. *Nature*, **316**, 552.

BEUTLER, B., MILSARK, I.W. & CERAMI, A. (1985*c*). Passive immunization against cachectin/tumour necrosis factor protects mice from the lethal effects of endotoxin. *Science*, **229**, 869.

BEUTLER, B. & CERAMI, A. (1986). Cachectin and tumour necrosis factor as two sides of the same biological coin. *Nature*, **320**, 584.

BLICK, M., SHERWIN, S.A., ROSENBLUM, M. & GUTTERMAN, J. (1987). Phase 1 study of recombinant tumour necrosis factor in cancer patients. *Cancer Res.*, **47**, 2986.

BRENNAN, M. (1977). Uncomplicated starvation versus cancer cachexia. *Cancer Res.*, **37**, 2359.

CERAMI, A., IKEDA, Y., LE TRANG, N., HOTEZ, P.J. & BEUTLER, B. (1985). Weight loss associated with an endotoxin-induced mediator from peritoneal macrophages. The role of cachectin (tumour necrosis factor). *Immunol. Lett.*, **11**, 173.

COSTA, G. (1963). Cachexia, the metabolic component of neoplastic diseases. *Prog. Exp. Tumour Res.*, **3**, 321.

FEINGOLD, K.R., GRUNFELD, C., MOSER, A.H., LEAR, S.R. & HUANG, B.-J. (1987). Tumor necrosis factor-alpha stimulates hepatic lipogenesis in the rat *in vivo*. *J. Clin. Invest.*, **80**, 184.

GARRATTINI, S., BIZZI, A., DONELLI, M.G., GUAITANI, A., SAMANIN, R. & SPREAFICO, F. (1980). Anorexia and cancer in animals and men. *Cancer Treatment Rev.*, **7**, 115.

KETTLEHUT, I.C., FIERS, W. & GOLDBERG, A.L. (1987). The toxic effects of tumour necrosis factor *in vivo* and their prevention by cyclo-oxygenase inhibitors. *Proc. Natl Acad. Sci. USA*, **84**, 4273.

LUNDHOLM, K., EDSTROM, S., KARLBERG, I., EKMAN, L. & SCHERSTEN, T. (1980). Relationship of food intake, body composition and tumor growth to host metabolism in non-growing mice with sarcoma. *Cancer Res.*, **40**, 2515.

NILSSON-EHLE, P. (1980). Lipolytic enzymes and plasma lipoprotein metabolism. *Ann. Rev. Biochem.*, **49**, 667.

OLIFF, A., DEFEO-JONES, D., BOYER, M. & 5 others (1987). Tumors secreting human TNF/cachectin induce cachexia in mice. *Cell*, **50**, 555.

ROBBINS, S.L. (1962). *Textbook of Pathology with Clinical Application*, 2nd edition. W.B. Saunders: Philadelphia.

SATOMI, N., SAKURAI, A. & HARANAKA, K. (1985). Relationship of hypoglycaemia to tumor necrosis factor production and antitumor activity: Role of glucose insluin and macrophages. *J. Natl Cancer Inst.*, **74**, 1255.

SEMB, H., PETERSON, J., TAVERNIER, J. & OLIVECRONA, T. (1987). Multiple effects of tumour necrosis factor on lipoprotein lipase *in vivo*. *J. Biol. Chem.*, **262**, 8390.

STRAIN, A.J. (1979). Cancer cachexia in man: A review. *Invest. Cell Pathol.*, **2**, 181.